# Developing a prediction model for disease‐free survival from upper urinary tract urothelial carcinoma in the Korean population

**DOI:** 10.1002/cam4.2382

**Published:** 2019-07-08

**Authors:** Sung Han Kim, Mi Kyung Song, Bumsik Hong, Seok Ho Kang, Byong Chang Jeong, Ja Hyun Ku, Ho Kyung Seo, Urothelial Cancer‐Advanced Research, Treatment (UCART) study group

**Affiliations:** ^1^ Department of Urology, Urologic Cancer Center National Cancer Center Goyang Korea; ^2^ Health Insurance Policy Research Institute, National Health Insurance Service Wonju Korea; ^3^ Department of Urology, Asan Medical Center University of Ulsan College of Medicine Seoul Republic of Korea; ^4^ Department of Urology Korea University Anam Hospital, Korea University College of Medicine Seoul Republic of Korea; ^5^ Department of Urology Samsung Medical Center, Sungkyunkwan University School of Medicine Seoul Republic of Korea; ^6^ Department of Urology Seoul National University Hospital, Seoul National University College of Medicine Seoul Republic of Korea

**Keywords:** nephroureterectomy, prediction model, prognosis, survival, urothelial carcinoma

## Abstract

**Background:**

In this study, we aimed to propose a validated prediction model for disease‐free survival (DFS) after radical nephroureterectomy (RNU) in a Korean population with upper urinary tract urothelial carcinoma (UTUC).

**Methods:**

We performed a retrospective review of 1561 cases of UTUC who underwent either open RNU (ONU, n = 906) or laparoscopic RNU (LNU, n = 615) from five tertiary Korean institutions between January 2000 and December 2012. Data were used to develop a prediction model using the Cox proportional hazards model. Prognostic factors were selected using the backward variable selection method. The prediction model performance was investigated using Harrell's concordance index (C‐index) and Hosmer‐Lemeshow type 2 statistics. Internal validation was performed using a bootstrap approach, and the National Cancer Center data set (n = 128) was used for external validation.

**Results:**

A best‐fitting prediction model with seven significant factors was developed. The C‐index and two Hosmer‐Lemeshow type statistics of the prediction model were 0.785 (95% CI, 0.755‐0.815), 4.810 (*P* = 0.8506), and 5.285 (*P* = 0.8088). The optimism‐corrected estimate through the internal validation was 0.774 (95% CI, 0.744‐0.804) and the optimism‐corrected calibration curve was close to the ideal line with mean absolute error = 0.012. In external validation, the discrimination was 0.657 (95% CI, 0.560‐0.755) and the two calibration statistics were 0.790 (*P* = 0.9397) and 3.103 (*P* = 0.5408), respectively.

**Conclusion:**

A validated prediction model based on a large Korean RNU cohort was developed with acceptable performance to estimate DFS in patients with UTUC.

## 1 BACKGROUND

Upper urinary tract urothelial carcinomas (UTUC) are relatively rare, accounting for 5%‐10% of urothelial tumors, and their incidence has slowly increased over the past 30 years.^1^ The current gold standard treatment of UTUC is radical nephroureterectomy (RNU) with bladder cuff excision. However, the 5‐year cancer‐specific mortality rates remain substantial at 20%‐30%,^2^ including 30%‐50% 5‐year overall survival (OS) rates in non–organ‐confined pT3‐4 disease and nodal metastatic disease.^3^ Thus, identifying biological and clinical factors that could optimize decision‐making through evidence‐ and risk‐based approaches is necessary. However, studies on UTUC are lacking, especially concerning prognosis in Korea.

Predicting disease‐free survival (DFS) may optimize follow‐up and improve post‐RNU management, such as adjuvant chemotherapy, which has been suggested without available level 1 evidence.^4^ Clinically, 3‐5‐year relative survival statistics are often used to measure cancer control and assess international comparisons.^2^, ^3^, ^5^ Nomograms have been built to integrate independent prognostic variables to better individualize and predict patient prognosis.^2^, ^6^ Initial cancer prognosis assessment at surgery helps to select post‐RNU therapy and follow‐up.

Large‐scale studies are necessary to increase a nomogram's accuracy and validate it with an additional patient cohort. Due to the rarity of UTUC and different surgical techniques with heterogenous patient cohorts, it is difficult to acquire sufficient data to explore characteristics of patients with UTUC. A nomogram was developed from Western UTUC cohorts,^2^, ^7^, ^8^, ^9^, ^10^ and the few Asian patient‐based nomograms have incorporated small cohorts of patients.^6^, ^8^, ^11^ Therefore, the aim of the current study was to determine a prediction model of DFS and OS of UTUC after RNU using a large, multicenter, Korean cohort, and to validate the nomogram model.

## 2 METHODS

### 2.1 Ethics approval and consent to participate

The protocol for this retrospective multicenter study was approved by the institutional review board of the National Cancer Center (NCC‐2016‐0040 and 2018‐0114‐0001), and complied with the principles of the Declaration of Helsinki. The requirement for written informed consent was waived based on the retrospective design. All patient data and records were anonymized before the analysis.

### 2.2 Patient population

We retrospectively reviewed data from 1561 patients with UTUC who underwent either open RNU (ONU, n = 906) or laparoscopic RNU (LNU, n = 615) from five tertiary Korean institutions (National Cancer Center, Asan Medical Center, Samsung Medical Center, Seoul National University Hospital, and Korea University Hospital) in the Urothelial Cancer‐Advanced Research and Treatment (UCART) study group between January 2000 and December 2012. Cases before 2000 were excluded to eliminate the potential bias of surgical inexperience with LNU. Exclusion criteria were: history of previous or concomitant radical cystectomy or bladder surgery, bilateral tumor, incomplete follow‐up records, and neoadjuvant chemotherapy. Age at surgery, gender, body mass index, American Society of Anesthesiologists score, previous bladder cancer, concomitant bladder cancer, tumor location, tumor stage, tumor grade, presence of lymphovascular invasion (LVI) or concomitant carcinoma in situ (CIS), lymph node status, receipt of adjuvant chemotherapy, follow‐up records, and oncologic outcomes were collected. The 1998 World Health Organization/International Society of Urologic Pathology consensus classification(13) for tumor grading and the 2010 American Joint Committee on Cancer/Union Internationale Contre le Cancer (Tumor‐Node‐Metastasis) classification for tumor staging were used.^12^, ^13^

### 2.3 Surgery and follow‐up

According to previously published papers from this original UCART dataset (published in *Cancer Research and Treatment,* January 2018), ONU or LNU was performed with/without lymphadenectomy; transperitoneal or retroperitoneal kidney dissection with the entire ureter length and adjacent bladder cuff segment were performed based on the surgeon's discretion. Adjuvant chemotherapy was administered according to pathologic stage to those who generally had non–organ‐confined disease (stage pT3‐4, N+).

Postoperative follow‐up was not standardized due to the retrospective multicenter design. Patients were generally evaluated every 3‐4 months during the first year post‐RNU, every 6 months during years 2‐5, and annually thereafter, including cystoscopy, serology, and urine tests (including urine cytology). Abdominal/chest computed tomography or magnetic resonance imaging was suggested annually or more often, depending on pathological stage.

### 2.4 Outcome

Disease‐free survival was defined as the duration between the date of RNU and the date of extravesical recurrence, disease progression, or death. To focus on early prognosis, and considering progressive UTUC, including 1‐year intravesical recurrence free survival and 5‐year cancer‐specific survival (CSS), 3‐year DFS was evaluated.^14^ Events over 3 years were censored and their durations were fixed at 3 years based on CT scans, and all‐cause deaths were defined as death events.

### 2.5 Statistical analysis

The population was classified into two data sets. One is the development set and the other is the external validation set. The development set is a random sample derived from a population of interest, and is used to develop a prediction model. The external validation set is used to perform external validation of the prediction model. It is independent of and differs in some aspects from the development set. In our study, the development set consisted of the multicenter data (n = 1561), and the National Cancer Center data set is considered the external validation set (n = 128). The subjects’ baseline characteristics according to the two sets are presented as frequencies with percentages. Cox proportional hazards model was used to develop a multivariable prediction model for 3‐year DFS. The candidate prognostic variables are presented in Table 1, and the variation inflation factor was calculated to explore multicollinearity between variables. The backward variable selection method with a type I error criterion of 0.05 was used to select factors significantly affecting 3‐year DFS. The prediction model performance was evaluated with respect to discrimination and calibration.^15^ Discrimination, indicating the ability to separate outcome categories, was measured using Harrell's concordance index (C‐index) with 95% confidence intervals: values range from 0.5 (classification by 1/2 probability) to 1.0 (perfect prediction). Calibration, indicating predicted risk reliability, was evaluated using the overall May and Hosmer goodness‐of‐fit testing, and Greenwood‐Nam‐D’ Agostino *χ* ^2^ statistic.^16^, ^17^ A smaller statistic indicates a predicted risk similar to the observed risk. Since the performance derived from the development set represents too optimistic an estimate, a bootstrap approach was employed to correct bias for internal validation. The bootstrap samples were derived with replacement from the development set, and the prediction model was developed in each bootstrap sample. Each model was evaluated both in the bootstrap sample and the development set. The difference in performance is the optimism, which is a kind of bias that implies overfitting. From 1000 bootstrap repetitions, we provided an optimism‐corrected estimate for performance measures. In particular, mean absolute error was calculated by the difference between the predicted values and the corresponding bias‐corrected calibrated values. External validation is important to investigate general applicability. The same methods described above were adopted to externally validate the prediction model. Ultimately, we generated a user‐friendly nomogram for clinical practice. We also applied the prediction model to 3‐year OS because OS is nested in DFS. We tried to investigate whether the linear predictor, calculated from the estimated coefficients and the observed values of the factors, predicts 3‐year OS. The results of statistical tests were two‐tailed and statistical significance was considered at *P* < 0.05. All analyses were performed using Statistical Analysis System (version 9.3, SAS Institute, Inc, Cary, NC) and R software (version 3.3.3; R Foundation for Statistical Computing, Vienna, Austria).

**Table 1:** Baseline characteristics in the development and the external validation sets

Variables	Development set (four center)	External validation set (NCC center)
Total N	1561	128
Operation		
ONU	896 (57.40)	81 (63.28)
LNU	665 (42.60)	47 (36.72)
Age, years		
≤55	332 (21.27)	21 (16.41)
56‐65	488 (31.26)	39 (30.47)
66‐75	530 (33.95)	47 (36.72)
≥76	211 (13.52)	21 (16.41)
Sex		
Male	1152 (73.80)	92 (71.88)
Female	409 (26.20)	36 (28.13)
BMI, kg/m^2^	24.20 (10.12‐48.23)	24.30 (15.80‐33.20)
ASA score		
1	390 (24.98)	28 (21.88)
2	1057 (67.71)	77 (60.16)
3	90 (5.77)	12 (9.38)
Unknown	24 (1.54)	11 (8.59)
Previous bladder cancer, n(%)		
No	1279 (81.93)	85 (66.41)
Previous bladder tumor Hx.	167 (10.70)	35 (27.34)
Concomitant bladder tumor Hx.	115 (7.37)	8 (6.25)
Tumor location		
Renal pelvis	749 (47.98)	8 (6.25)
Ureter	641 (41.06)	2 (1.56)
Both renal pelvis and ureter	171 (10.95)	118 (92.19)
Tumor grade		
Low grade	481 (30.81)	31 (24.22)
High grade	1048 (67.14)	88 (68.75)
Unknown	32 (2.05)	9 (7.03)
Pathological T stage		
pTa/pT1‐2	908 (58.17)	63 (49.22)
pT3‐4	638 (40.87)	63 (49.22)
CIS	15 (0.96)	2 (1.56)
Pathological N stage		
pNx	825 (52.85)	81 (63.28)
pN0	615 (39.40)	38 (29.69)
pN1	121 (7.75)	9 (7.03)
Concomitant LVI	331 (21.20)	36 (28.13)
Concomitant CIS	217 (13.90)	22 (17.19)
Follow‐up duration (months)	39.68 (1.0‐184.37)	41.64 (1.0‐151.8)

Abbreviations: CIS, concomitant carcinoma in situ; LVI, lymphovascular invasion.

## 3 RESULTS

### 3.1 Population characteristics

**Figure 1:**
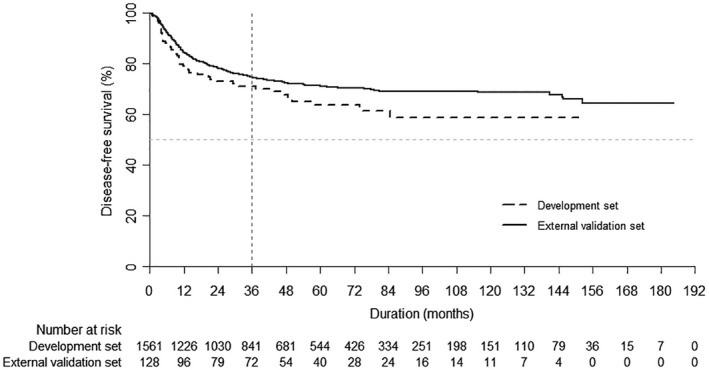
Kaplan‐Meier curves for disease‐free survival in each set

The baseline characteristics of the development and external validation sets are presented in Table 1; the Kaplan‐Meier DFS curve is shown in Figure 1. In the development set, there were 51 (12.23%) events of disease progression after 36 months, with a 74.82% 3‐year DFS rate (95% CI, 72.56%‐77.07%). In the external validation set, there were eight events (18.60%) of disease progression after 36 months, with a 71.04% 3‐year DFS rate (95% CI, 62.91%‐79.17%).

### 3.2 Model development

Table 1 shows the clinicopathological variables used for model development. The variation inflation factors to investigate multicollinearity between variables confirmed no multicollinearity (all VIF < 2). After backward selection, a best‐fitting prediction model was developed, consisting of seven factors: age group, ASA score, previous bladder cancer, tumor grade, pathological T/N stage, and LVI (Table 2). Although part of the categories for each variable were not significant, the overall effect of each variable, that is, type 3 test, was significant (*P* < 0.05), except for ASA score (*P* = 0.068).

**Table 2:** The best‐fitting prediction model from the backward selection method

Variables	Coefficient (SE)	HR (95% CI)	*P*‐value
Age, years			
≤55		1 (Ref)	
56‐65	0.054 (0.160)	1.056 (0.772‐1.445)	0.7337
66‐75	0.456 (0.153)	1.577 (1.168‐2.130)	0.0029
≥76	0.432 (0.196)	1.540 (1.049‐2.263)	0.0277
ASA score			
1		1 (Ref)	
2	−0.253 (0.125)	0.776 (0.608‐0.991)	0.0422
3	−0.436 (0.285)	0.647 (0.370‐1.130)	0.1261
Unknown	0.622 (0.356)	1.863 (0.927‐3.745)	0.0807
Previous bladder cancer			
No		1 (Ref)	
Previous bladder tumor Hx.	0.378 (0.156)	1.459 (1.075‐1.982)	0.0155
Concomitant bladder tumor Hx.	0.420 (0.186)	1.522 (1.057‐2.190)	0.0238
Tumor grade II			
Low grade		1 (Ref)	
High grade	0.809 (0.184)	2.245 (1.567‐3.218)	<0.0001
Unknown (II 포함)	0.456 (0.567)	1.577 (0.519‐4.794)	0.4217
Pathological T stage			
pTa/pT1‐2		1 (Ref)	
pT3‐4	1.108 (0.131)	3.029 (2.344‐3.915)	<0.0001
CIS	−0.581 (1.075)	0.559 (0.068‐4.601)	0.5889
Pathological N stage			
pNx		1 (Ref)	
pN0	−0.072 (0.123)	0.930 (0.731‐1.183)	0.5555
pN1	0.812 (0.150)	2.253 (1.679‐3.023)	<0.0001
Concomitant LVI			
No		1 (Ref)	
Yes	0.683 (0.118)	1.980 (1.570‐2.497)	<0.0001

Abbreviations: CIS, concomitant carcinoma in situ; LVI, lymphovascular invasion.

### 3.3 Performance results, internal, and external validation

**Figure 2:**
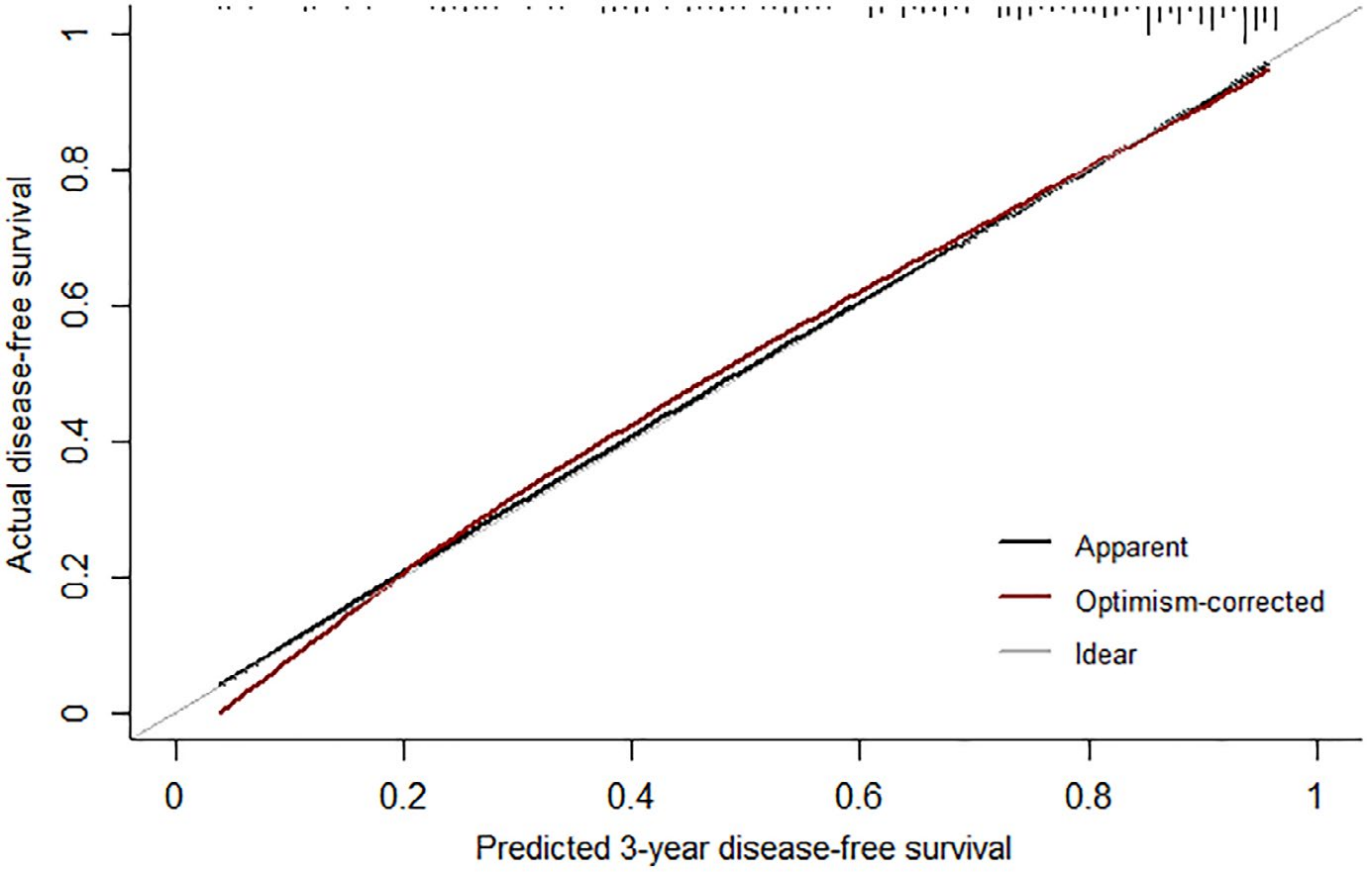
Calibration plot for the prediction model

The performance results of the prediction model are shown in Table 3. The estimated probability from the prediction model was similar to the observed probability. Figure 2 shows that the optimism‐corrected loss was close to the ideal 45° line, although slightly different from the apparent calibration curve (mean absolute error = 0.012). For external validation, the prediction model and external validation dataset were used to calculate risk. We then evaluated the performance to identify how well the calculated risk predicts the observed 3‐year DFS. Although the C‐index decreased somewhat compared to the apparent or internal validation results, it showed moderate predictive performance (Table 3).

**Table 3:** Performance of the prediction model

	Development	External validation
Discrimination ability		
C‐index (95% confidence interval)	0.785 (0.755‐0.815)	0.657 (0.560‐0.755)
Optimism‐corrected estimate (95% confidence interval)	0.774 (0.744‐0.804)^a^	—
Calibration ability		
Goodness‐of‐fit by May and Hosmer (*P*‐value)	4.810 (0.8506)	0.790 (0.9397)
Greenwood‐Nam‐D'Agostino statistics (*P*‐value)	5.285 (0.8088)	3.103 (0.5408)

— Optimism from 1000 bootstrapping repetitions = 0.011.

a Assuming the same SE applies as estimated for model development.

### 3.4 Nomogram for clinical application

**Figure 3:**
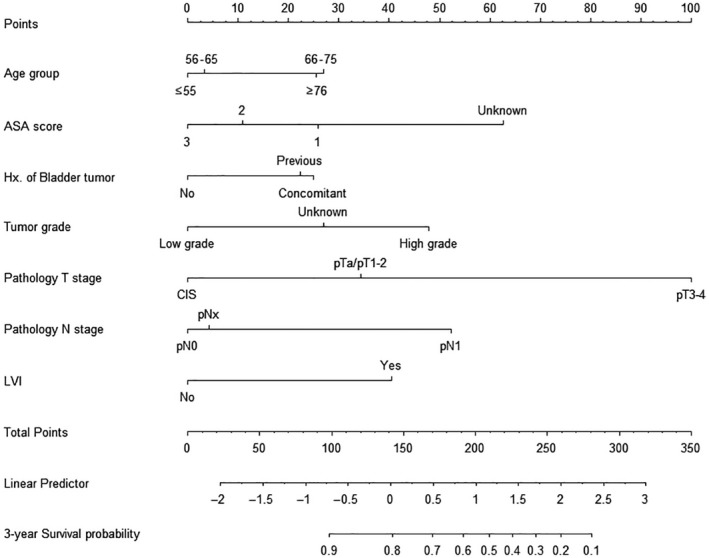
Nomogram for predicting the risk of 3‐y disease‐free survival

The user‐friendly prognostic nomogram for predicting for 3‐year DFS is shown in Figure 3. The relative risk factors are assigned specific points on a 0‐100 scale according to their regression coefficients. The sum of the points drawn on the “Total Points” line corresponds to the probability of 3‐year DFS, represented at the bottom. For example, for a 78‐year‐old subject with ASA score 2, unknown tumor grade, pathological T stage CWAS, no lymph node dissection, and no LVI, the total number of points is 67.63, and the corresponding 3‐year DFS probability is greater than 0.9.

### 3.5 Three‐year OS

Three‐year OS was assessed to determine whether the risk from the prediction model of 3‐year DFS accounted for 3‐year OS. The discrimination abilities were 0.775 (95% CI, 0.740‐0.809) in the development set and 0.693 (95% CI, 0.578‐0.808) in the external validation set, and all had acceptable performance. For calibration ability, the results showed similar predicted and observed values in the development set (statistic = 14.171, *P* = 0.12), but the results were meaningless in the external validation set (statistic = 10.571, *P* = 0.032).

## 4 DISCUSSION

Prediction models estimate prognosis using various baseline and intraoperative clinicopathological findings from the time of UTUC diagnosis after surgical resection of UTUC and prior to implementation of chemotherapy. The rarity and the dismal prognosis of UTUC have made it difficult to determine an efficient prediction model with significant predictive prognostic factors. Since the early 2010s, various prediction models with nomograms have been developed to predict the IVRFS, DFS, CSS, and OS after RNU in UTUC patients; however, most have been based on Western patients.^2^, ^6^, ^7^, ^8^, ^9^, ^10^, ^18^, ^19^ Only a few Asian prediction models have been developed to predict prognostic and functional outcomes after RNU in UTUC.^6^, ^8^, ^11^

Because the prediction model was based on the enrolled subjects’ clinicopathological parameters, the geography, and ethnicity of different cohorts might influence the prognostic outcome of the model(21).^20^ It is therefore necessary to analyze significant predictive risk factors of prognosis from people of the same geographical background and to incorporate the prediction model based on cohorts from same ethnic backgrounds. A population‐based US study found that African‐American patients with UTUC had a shorter survival than other ethnic groups,^1^ and Chinese people had a higher incidence of UTUC due to their lifetime intake of herbal tea.^20^ Our prediction model was based on Korean patients with UTUC after RNU. Four prediction models based on clinicopathological factors already exist from cohorts with similar ethnic backgrounds: one Japanese model and one Chinese model of postoperative renal insufficiency,^11^ one Chinese model of postoperative complications,^12^ and one Korean model of survival prognosis.^6^, ^8^ However, the former 2 did not consider survival prognosis, and the remaining two models that did were developed and validated with small cohorts.

Accordingly, the prediction model in this study is the first to incorporate a large cohort of East Asian patients with UTUC after RNU with acceptable validation and power comparable to that of Western prediction models.^7^, ^13^, ^21^ Among many Western prediction models with various parameters, only some have had large development and validating sets of patients focused on either 3‐ or 5‐year CSS with an accuracy of 0.7‐0.8,^2^, ^3^, ^9^, ^18^ similar to this study. Given the rarity of UTUC and difficulty of long‐term follow‐up because of poor prognosis, this study's strengths are that it provides a useful prediction model in Asian UTUC patients who underwent RNU and considers diverse parameters, whereas the only two existing Asian prediction models did not have similarly large cohorts for the development and validation sets, but instead used small cohorts to evaluate IVRFS and CSS.^6^, ^11^

This study considered 3‐year DFS as a prognostic outcome^14^ because the intravesical recurrence after RNU was approximately 20%‐50% within 1‐1.5 years,^22^, ^23^ and the OS or CSS was estimated at 3‐ or 5‐years.^2^, ^6^, ^10^ Therefore, the 3‐year DFS comprised local recurrence, cancer‐specific, and non–cancer‐specific death, but not intravesical recurrence. With this background, we developed the prediction model for 3‐year DFS based on multicenter data. The significant variables of the prediction model were validated previously (*Cancer Research and Treatment*, accepted in March 2018). Increased age, previous bladder tumor history, higher tumor grade, higher pathologic T and N stages, and concomitant presence of LVI are known poor prognostic factors.^1^, ^2^, ^3^, ^5^, ^6^, ^7^, ^8^, ^9^, ^10^, ^11^, ^18^, ^24^

However, this study found that an increased ASA score was a favorable risk factor, which is contradictory to previous results of ASA as a negative risk factor for survival.^25^ This contradictory result might be explained by selection bias due to the characteristics of cohorts including group 3 ASA patients with lower tumor burdens, who might receive RNU successfully. Patients with higher comorbidities and higher ASA scores were more likely to undergo chemotherapy instead of RNU because of surgical morbidity delaying adjuvant chemotherapy. Those selected patients with high ASA scores who underwent RNU likely had a small tumor volume and early‐stage cancer, so their DFS might have been significantly better than that of patients with ASA ≤ 2.

The insignificant sex, CIS, BMI, and surgical modality of this study have been indicated as predictive factors for prognosis in other studies.^3^, ^8^, ^26^ In one study, female sex was associated lower IVRFS after RNU in UTUC (HR 0.812, 95% CI 0.673‐0.981, Table S1 for IVRFS).^19^ However, similar to our results, another study reported no significant association between sex and CSS (HR 1.050, 95% CI 0.841‐1.310, Table S1 for OS).^2^ Some studies have shown that CIS is a significant adverse prognostic factor,^3^ whereas others have not.^27^, ^28^ As for the BMI, different version existed on the prognostic significance of survival in UTUC that obese UTUC patients had significantly worse CSS than the other three BMI groups (*P* = 0.031). The association between surgical technique, such as laparoscopic RNU, and survival outcome has been debated, and several meta‐analyses have shown no significant differences in oncological outcome including IVRFS, CSS, OS, and metastasis rates based on surgical technique.^28^

The prediction model in this study had a moderate performance in internal validation. The calibration statistics indicated that the model was reliable (*P* > 0.05) using an external validation set. Our model can be used in clinical practice by application of the nomogram. Namely, the 3‐year DFS rate can be estimated by exponentially multiplying the linear predictor value, which corresponds to the sum of the points assigned to each variable, to the 3‐year cumulative survival rate of 0.8043; S(3, X) = [0.8043]^exp(the value of linear predictor)^.

This study had several limitations, including a retrospective study design, collection biases, multicentric heterogeneity of standardized surgical procedures, and postoperative therapeutic decisions (ie, the extent of lymph node dissection and adjuvant chemotherapy protocol), and the absence of multiple other known prognostic variables, such as intraoperative parameters and baseline social lifestyle and comorbidities. Although this is the first large study of Asian patients with UTUC, a future study with an even larger multi‐institutional database and all potential parameters of prognosis will be planned to improve the discriminatory ability of the predictive model for UTUC.

## 5 CONCLUSIONS

A validated prediction model with an acceptable performance for clinical use was developed using clinicopathological variables from large Asian RNU cohorts. For patients with UTUC, this model could help estimate prognosis and select appropriate treatment.

## CONFLICT OF INTEREST

All authors declare that they have no conflict of interest.

## DATA AVAILABILITY STATEMENT

The datasets used and/or analyzed during the current study available from the corresponding author (Ho Kyung Seo, seohk@ncc.re.kr) on reasonable request. The IRB and ethical committee of the National Cancer Center (in Korea) will review the requests because of the patients' information. After the approval of the committee with confirmation of the reasonable requests, the dataset will be freely available. The other contact e‐mail besides the corresponding author's e‐mail is irb@ncc.re.kr
