# Supplementary material for: Developing a prediction model for disease‐free survival from upper urinary tract urothelial carcinoma in the Korean population
Source: Cancer Med. 2019 Jul 8;8(11):4967–75. doi: 10.1002/cam4.2382 (PMC6718545; doi:10.1002/cam4.2382)
Supplement: Supplementary file 1 [file CAM4-8-4967-s001.docx]

**Supplementary Table 1**. Comparative description of previous nomogram studies

| Authors | Number of patients | Participants’ year | Significant variable | Prognostic outcome | Region v | Result |
| --- | --- | --- | --- | --- | --- | --- |
| Edhaie et al.([7](#_ENREF_7)) | 253 from single institution | 1995-2008 | Age, gender, ASA, smoking, CIS,multifocality, tumor grade, pT, Nstage, LN status | 5yr RFS, CSS | The USA (New York) | RFS -78.3%  CSS- 78% |
| Seisen et al.([9](#_ENREF_9)) | 2233 from 44 institutions  (Develop. 1563/ Validation 670) | 1987-2010 | Age, gender, pT,N stage, tumor grade, location, LVI, tumor architecture, CIS | 5yr CSS | Europe | CSS 5yr-75.7%  Accuracy 0.81 |
| Yates et al([10](#_ENREF_10)) | 667 from 21 institutions, (Develop 397, validate 270) | 1995-2010 | Location, grade, age, pT,N stage | 5yr CSS | France | CSS 5yr-76% ,accuracy 0.78 |
| Raman et al.([8](#_ENREF_8)) | 731 from 8 insitutions | 2002-2010 | Age, ECOG, CKD, CCI | 30day postop complication | The USA and France | 30 day postop complication 72.2% |
| Jeldes et al([19](#_ENREF_19)) | 5918 from SEER database (Develop 2959/validate 2959) | 1988-2006 | Age, pT, N stage, tumor grade | 5yr CSS | The USA | CSS 5yr- 75.4%,  Accuracy 0.773 |
| Roupret et al([2](#_ENREF_2)) | 3387 from 2 largest multicenter dataset (Develop 2371/validate 1016) | ??-2011 | Age, pT,N stage, tumor architecture, LVI | 5yr CSS | France | CSS 5yr-732.7%,  Accuracy 0.8 |
| Ku JH et al.([6](#_ENREF_6)) | 323 from single insitution | 1991-2010 | Age, location, bladder cuffing modality, pT,N stage, tumor grade, surgical margin | 3,5yr-CSS | Korea | CSS 3yr/5yr- 71.6%/71.8%,  Accuracy 0.742 |
| Feng D et al.([11](#_ENREF_11)) | 666 from 27 insitutions | 2000-2010 | Age, preop. eGFR, location, HN, multifocality, tumor size | Postop. renal insufficiency | China | 30 day postop complication |
| Ishioka et al.([26](#_ENREF_26)) | 754 from 13 insituions | 1995-2010 | Gender, pT stage, tumor architecture, LVI | 1yr/5yr IVRFS | Japan | IVRFS 1yr/5yr- 15%/29%;  Accuracy 0.62 |
| Xylinas et al. ([20](#_ENREF_20)) | 1839 (develop 1261/ validate 578) |  | age, gender, tumor location, surgical modality, bladder cuff methods, previous bladder tumor history, pT, N stage, CIS | 3,6,9,12,18,24,36 months IVRFS | North America | IVRFS 24mos- 15%  Accuracy 0.678/ 0.69 |
| This study | 1689 from 5 insitutions (develop 1561/validate 128) | 2000-2012 | Age, ASA score, tumor grade, LVI, previous bladder tumor history, pathologic T and N stage | 3yr-disease progression free survival | Korea | 3yr-disease progression free survival 78.5%,  Accuracy 0.657 |
